# Inhibition of CXCR2 plays a pivotal role in re-sensitizing ovarian cancer to cisplatin treatment

**DOI:** 10.18632/aging.203074

**Published:** 2021-05-26

**Authors:** Taciane Barbosa Henriques, Diandra Zipinotti dos Santos, Isabella dos Santos Guimarães, Nayara Gusmão Tessarollo, Paulo Cilas Morais Lyra-Junior, Patricia Mesquita, Diana Pádua, Ana Luisa Amaral, Bruno Cavadas, Luisa Pereira, Ian Victor Silva, Raquel Maria da Silva Graça Almeida, Leticia Batista Azevedo Rangel

**Affiliations:** 1 Biotechnology Program/RENORBIO, Health Sciences Center, Federal University of Espirito Santo, Espirito Santo, Brazil; 2 Translational Research Laboratory/Division of Clinical Research and Technological Development/National Cancer Institute (INCa), Rio de Janeiro, Brazil; 3 Instituto de Investigação e Inovação em Saúde (i3S), University of Porto, Porto, Portugal; 4 Institute of Pathology and Molecular Immunology (IPATIMUP), University of Porto, Porto, Portugal; 5 Department of Morphology, Health Sciences Center, Federal University of Espirito Santo, Espirito Santo, Brazil; 6 Biochemistry and Pharmacology Program, Federal University of Espirito Santo, Espirito Santo, Brazil; 7 Pharmaceutical Sciences Department, Health Sciences Center, Federal University of Espirito Santo, Espirito Santo, Brazil; 8 Biology Department, Faculty of Sciences, University of Porto, Porto, Portugal

**Keywords:** chemoresistance, high grade serous ovarian cancer, CXCR2, tumor microenvironment

## Abstract

cDNA microarray data conducted by our group revealed overexpression of CXCL2 and CXCL8 in ovarian cancer (OC) microenvironment. Herein, we have proven that the chemokine receptor, CXCR2, is a pivotal molecule in re-sensitizing OC to cisplatin, and its inhibition decreases cell proliferation, viability, tumor size in cisplatin-resistant cells, as well as reversed the overexpression of mesenchymal epithelium transition markers. Altogether, our study indicates a central effect of CXCR2 in preventing tumor progression, due to acquisition of cisplatin chemoresistant phenotype by tumor cells, and patients’ high lethality rate. We found that the overexpression of CXCR2 by OC cells is persistent and anomalously confined to the cellular nuclei, thus pointing to an urge in developing highly lipophilic molecules that promptly permeate cells, bind to and inhibit nuclear CXCR2 to fight OC, instead of relying on the high-cost genetic engineered cells.

## INTRODUCTION

Ovarian cancer (OC) is the eighth-leading cause of cancer-related deaths amongst women. In 2018, over 295,000 new cases, and 180,000 deaths were OC-associated [1]. Invasive epithelial ovarian cancer (EOC) is classified in five prevalent subtypes that originate from both secretory epithelial cells of the distal fallopian tube or from the ovarian epithelium and other tissues [2–7]. Mutational analysis of EOC samples has subdivided the serous OC in type I (less aggressive tumors, including low-grade serous, endometrioid, mucinous, and clear cells carcinomas, mutated for KRAS, BRAF and PI3K) and type II (highly aggressive tumors characterized by TP53 mutation [8–13], and high incidence of double-strand DNA break repair pathways [13–16]. EOC features justify, at least partially, the initial satisfactory response to platin and taxane derivate compounds therapy. Nonetheless, approximately 80% of the patients experience disease recurrence due to chemoresistance [17].

Chemokines secreted in tumor microenvironment (TME), as CXCL2 and CXCL8, correlate to chemoresistance [18–21]. In agreement, cDNA microarray data obtained from NAC-1 knockdown (KD) in high grade serous ovarian cancer (HGSOC) cells, a molecule crucial for the disease development and progression, revealed CXCL2 and CXCL8 secretion [18]. Moreover, these chemokines exert autocrine effect on OC cells by binding to CXCR2 and promoting chemoresistance (manuscript in preparation). CXCR2 activation is associated with cell proliferation, angiogenesis, metastasis and chemoresistance in melanoma, colon, lung and ovarian cancers [19–24].

Herein, we have shown that CXCR2 is crucial for the acquisition of cisplatin chemoresistant phenotype by OC cells, hence introducing a novel potential target against OC.

## RESULTS

### OC pan resistant ACRP cells were generated from the parental sensitive lineage A2780

**Figure 1:**
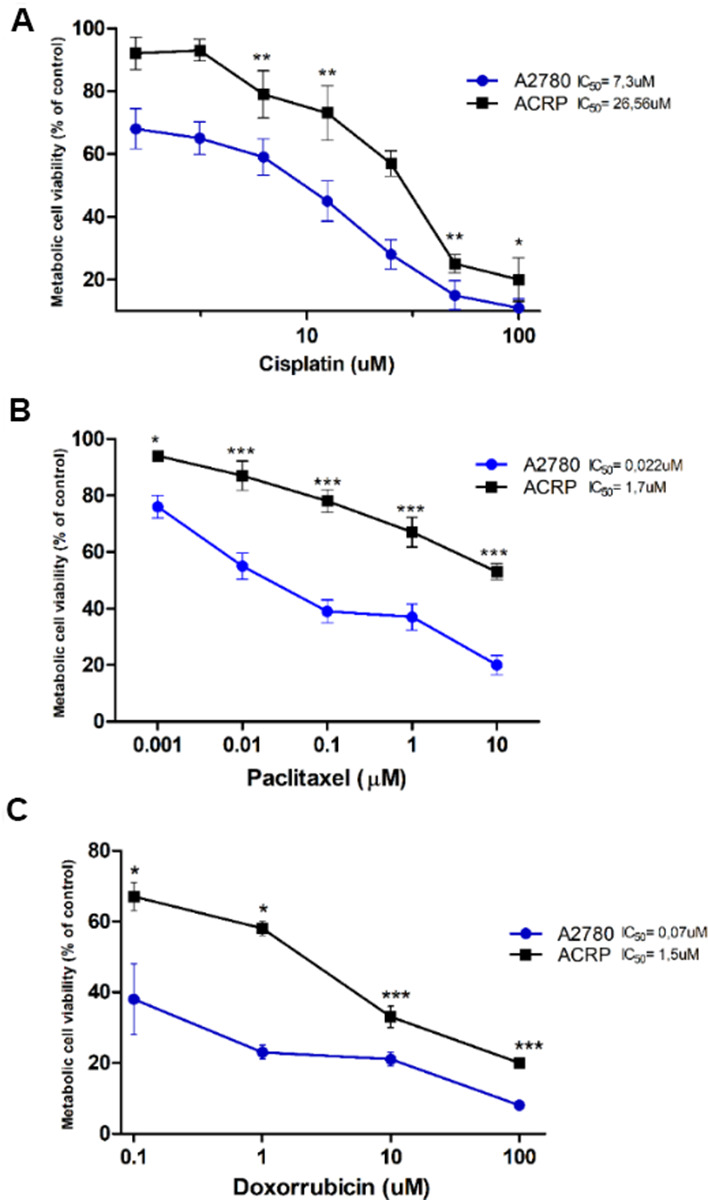
**Generation of a pan-resistant ovarian cancer (OC) cells (ACRP) from its parental sensitive counterpart (A2780).** (**A**) ACRP is 3.64-fold more resistant to cisplatin (1.5uM - 100uM) than A2780. (**B**) ACRP is 77.27-fold more resistant to paclitaxel (1 nM - 10 μM) than A2780. (**C**) ACRP is 21.42-fold more resistant to doxorubicin (0.1uM - 100uM) than A2780. Estimated IC_50_ for drugs in tested lineages were calculated by MTT assay, following 24h of cells treatment with each drug within the aforementioned concentration ranges of drugs, which correlate to their circulating concentration in EOC patients. Results are expressed as percentage of control (untreated cells) as mean ± SD. Statistical analyses of the results were done by two-way ANOVA followed by Bonferroni *post- test*. *p<0.01, **p<0.005, ***p<0.001. N=3.

ACRP cells were generated from its parental counterpart A2780 lineage, following chronic exposure to cisplatin (1μM to 10 μM). MTT method was used to estimate IC_50_ for cisplatin, paclitaxel and doxorubicin that were 3.64-fold (p<0.005) (Figure 1A), 77.27-fold (p<0.001) (Figure 1B), and 21.42-fold (p<0.001) (Figure 1C) higher in ACRP than in A2780, respectively, thus proving that the ACRP has emerged as a pan-resistant lineage. Cross-resistance to antineoplastic drugs, as observed in our *in vitro* study model, supports the need to elucidate the mechanisms of chemoresistance acquisition by OC cells and chemotherapy failure.

### CXCR2 is overexpressed and modulates the expression of CXCL2 and CXCL8 in ACRP cells

**Figure 2:**
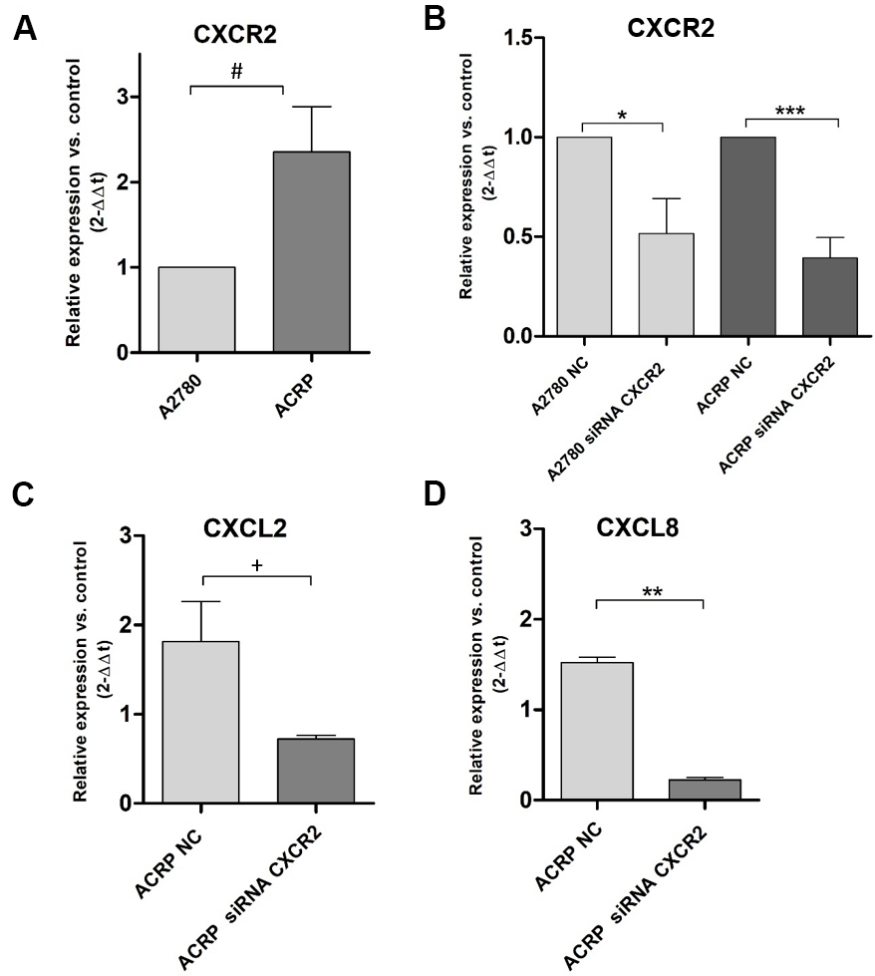
**CXCR2 is overexpressed and modulates the expression of CXCL2 and CXCL8 in ACRP cells and CXCR2 KD.** Transcript expression of CXCR2, CXCL2 and CXCL8 was investigated by qRT-PCR, following the protocol described in Material and Methods session, in both wild-type and CXCR2 KD (siRNA CXCR2, 10μM) A2780 and ACRP OC cells. (**A**) ACRP expressed 2.3-fold more CXCR2 than A2780 (^#^p=0.0342). (**B**) CXCR2 KD lead to lower expression of CXCR2 in both cell lines, however 1.3-fold less in ACRP (***p=0.001) than in A2780 (*0.0246). (**C**) CXCL2 expression was 2.5-fold lower in ACRP CXCR2 KD than in ACRP NC (^+^p=0.0362). (**D**) CXCL8 expression decreased by 6.5-fold comparing ACRP CXCR2 KD to ACRP NC (**p=0.0025). Differential gene expression was presented as relative expression of each gene of interest compared to control, after normalization by the expression of the housekeeping gene GAPDH and calculated by the 2^-ΔΔCt^ method. Data were analyzed by unpaired t-Student test (p<0.05). N=3.

Our previous data from cDNA microarray assays following NAC-1 KD in HGSOC cells [18] demonstrated that CXCL2 and CXCL8 are secreted in TME and can be correlated to chemoresistance (manuscript in preparation). We investigated this novel mechanism possibly underlying cisplatin resistance in EOC clinics. qRT-PCR experiments were run to evaluate CXCR2, CXCL2 and CXCL8 expression in ACRP cells. Our results revealed overexpression of CXCR2 in ACRP when compared to A2780 by 2.3-fold (p=0.034) (Figure 2A). Then, we downregulated the expression of CXCR2 in A2780 (p=0.0246) and in ACRP (p=0.001) by approximately 50% when compared to the negative control (NC) cells (Figure 2B). Further exploring the impact of CXCR2 expression in OC chemoresistance, we found that ACRP CXCR2 KD cells expressed 2.5-fold less CXCL2 (p=0.0362) (Figure 2C), and 6.5-fold less CXCL8 in comparison to NC cells (p=0.0025) (Figure 2D). Expression of CXCL2 and CXCL8 was not significantly modified in A2780 CXCR2 KD. Altogether, our results suggest the occurrence of an intricate CXCL2/CXCL8-CXCR2 axis in the modulation of cisplatin sensitivity by OC cells, thus corroborating with an important role of TME and a potential autocrine effect of CXCL2/CXCL8 on CXCR2 expressed by tumor cells.

### CXCR2 comprises nuclear expression in OC cells

**Figure 3:**
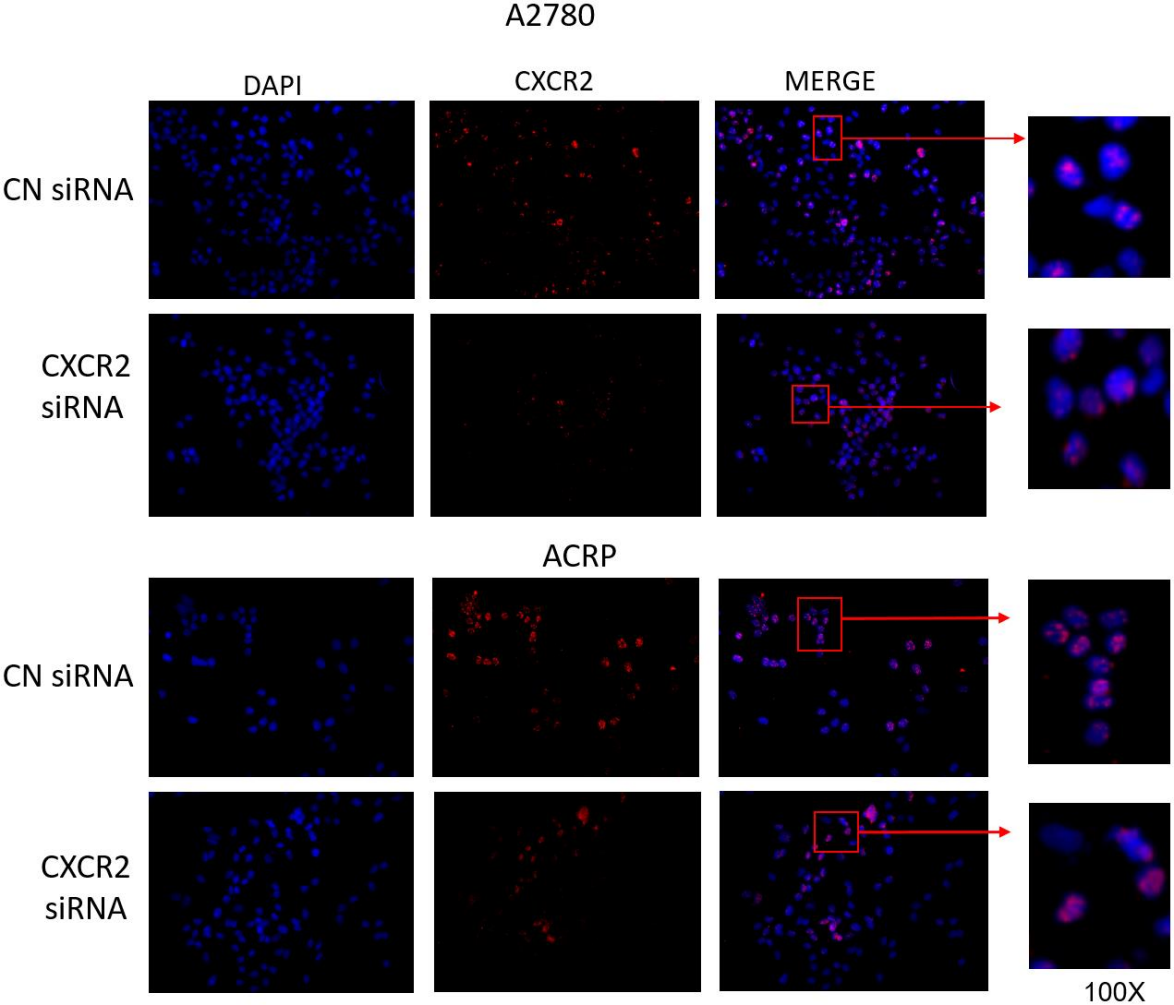
**CXCR2 comprises persistent nuclei anomalous expression cisplatin resistant OC cells.** Immunofluorescence assays were performed to investigate cellular localization of CXCR2 expression in OC cells. CXCR2 antibody (red) whereas cellular nuclei were stained with DAPI (blue); images where further merged to facilitate cellular localization analysis. Cells were plated at same density (left column of Figure). As previously demonstrated, CXCR2 was overexpressed in ACRP cells in comparison with A2780 cells (middle column of Figure). N=3. Images were acquired under 10x magnification.

Motivated by the aforementioned results, we decided to characterize the expression of CXCR2 in OC cells with regard to its cellular localization. To do so, we conducted immunofluorescence experiments with A2780 and ACRP, NC and CXCR2 KD (Figure 3, left column; nuclei stained with DAPI; blue). Treatment of cells with anti-CXCR2 antibody (red) revealed the overexpression of CXCR2 by ACRP in comparison with A2780 (middle column, Figure 3). CXCR2 was confined to the nuclei of A2780 and ACRP NC (Figure 3, right column). Nuclei expression of CXCR2 was sustained exclusively in ACRP CXCR2 KD (Figure 3, right column). Although the biological relevance of our observation remains unclear, it is imperative to point that chemoresistance to cisplatin is likely caused or aggravated by the sustained anomalous expression of CXCR2 in OC cells nuclei. In any event, we, herein, introduce a novel mechanism contributing to cisplatin chemoresistance in OC cells.

### CXCR2 overexpression correlates to patients’ low overall survival with primary EOC

**Figure 4:**
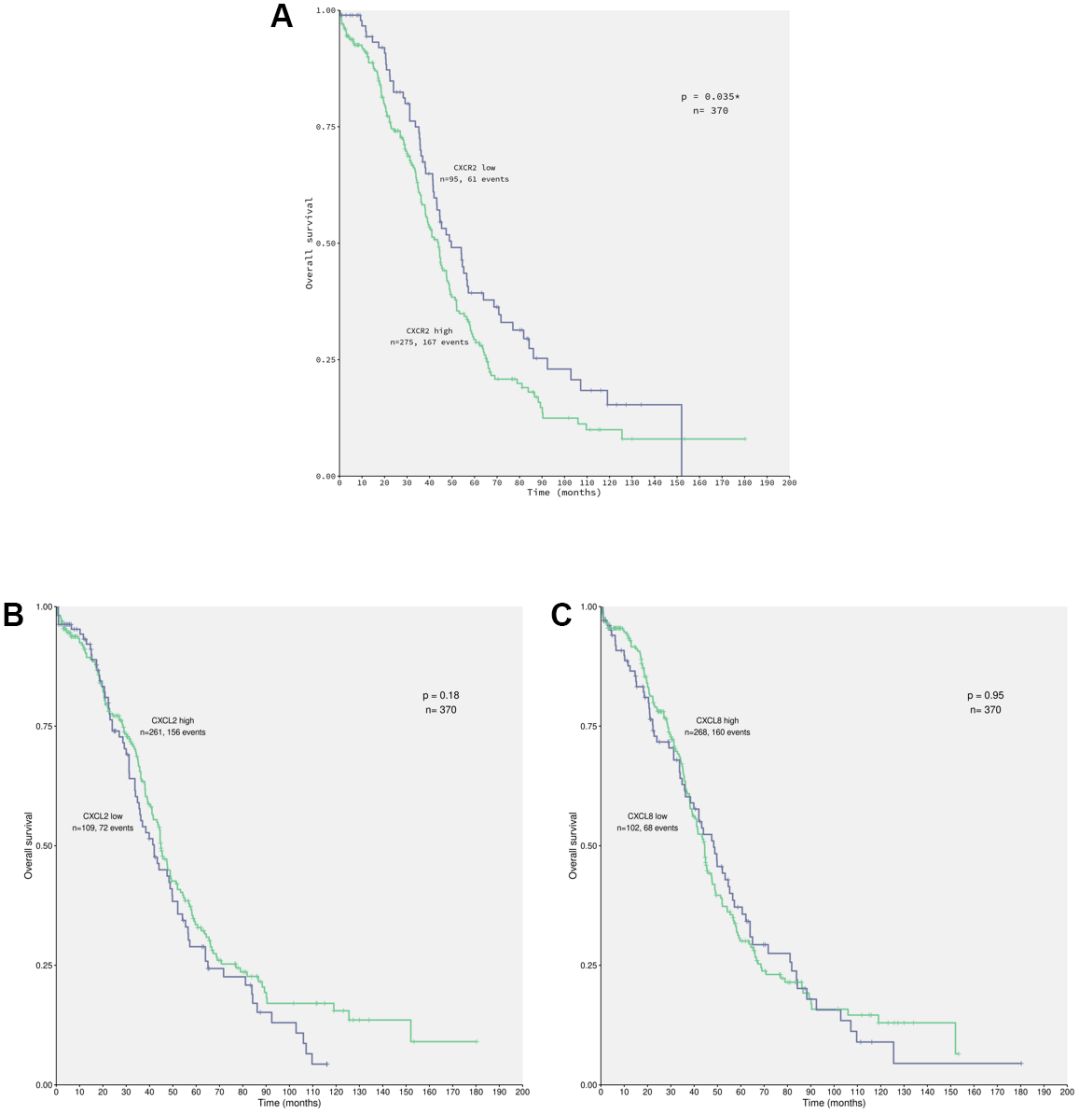
**CXCR2 overexpression correlates to patients’ low overall survival carrying primary EOC.** Data from 370 patients diagnosed with serous EOC were obtained from TCGA database for in silico analysis of patients’ overall survival (OS) in relation with the chemokines of interest expression by cancer cells, using the Kaplan-Meier statistical method. (**A**) Correlation of EOC patients, which tumor cells express low (n=95) or high (n=275) levels of CXCR2 to patients’ OS. Note that patients that carry EOC expressing high levels of CXCR2 had lower overall survival rate in comparison to the ones with low levels of the chemokines receptor (p=0.035). Moreover, overexpression of CXCR2 was identified in 74.32% of the studied patients. (**B**) Correlation of EOC patients, which tumor cells express low (n=109) or high (n=261) levels of CXCL2 to patients’ overall survival. No significant difference were observed with regard to CXCL2 expression by cancer cells and patients’ overall survival rate (p=0.18). However, overexpression of CXCL2 was seen in 70.54% of patients. (**C**) Correlation of EOC patients, which tumor cells express low (n=102) or high (n=268) levels of CXCL8 to patient’s overall survival. No significant differences were observed confronting CXCL8 expression by cancer cells to patients’ overall survival rate (p=0.95). Nonetheless, overexpression of CXCL8 was detected in 72.43% of patients. Long rank test was performed to analyse statistical difference amongst the parameters investigated.

Secretion of CXCL2 and CXCL8 in the conditioned medium of ACRP potentially act autocrinally on CXCR2 expressed by OC cells (manuscript in preparation). To evaluate the correlation between the expression of CXCR2 and EOC patients’ OS (overall survival), data from 370 patients diagnosed with primary EOC were obtained from TCGA database (Figure 4). Overexpression of CXCR2 was detected in 74.32% of the patients. EOC patients whose tumor cells expressed high levels of CXCR2 had lower OS compared with the cases expressing low levels of CXCR2 (n=0.035) (Figure 4A). No significant statistical differences were observed in OS of patients with EOC cells expressing low (n=109) or high (n=261) levels of CXCL2 (p=0.18) neither low (n=102) or high (n=268) levels of CXCL8 (p=0.95) (Figure 4B, 4C). Nonetheless, 70.54% and 72.43% of EOC cells overexpressed CXCL2 and CXCL8, respectively. Our results suggest that EOC cells can potentially secret CXCL2 and CXCL8 to TME that act autocrinally on tumor cells CXCR2, conferring the poor prognosis of the disease that is inferred by low patients’ OS. We postulate that CXCR2 emerges as a prognostic marker and a potential therapeutic target of EOC.

### CXCR2 enhances OC cell proliferation and cellular viability

**Figure 5:**
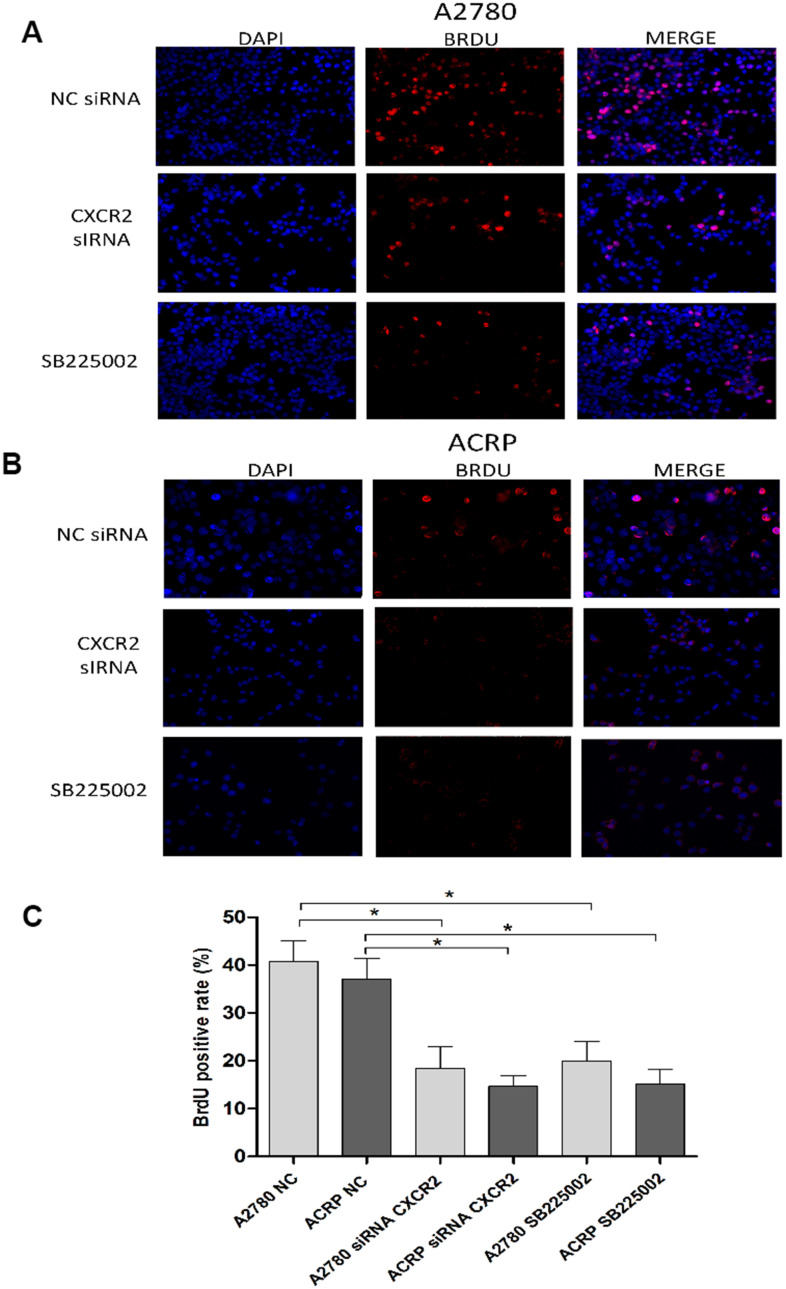
**CXCR2 enhances OC cell proliferation.** BrdU immunofluorescence assays were conducted to investigate the role of CXCR2 on OC cell proliferation. In brief, cells were plated at the same density and fixed, as previously reported. Antibody against the marker of cell proliferation, BrdU, was conjugated with PE (red), whereas cells nuclei were stained with DAPI (blue). Finally, both staining conditions were merged for better visualization of the studied phenomenon. (**A**) A2780 cells were either transfected with empty vector for control (NC), siRNA CXCR2 (10μM) or treated with the CXCR2 antagonist SB225002 (1ug/ml). (**B**) ACRP cells were either transfected with empty vector for control (NC), siRNA CXCR2 (10μM) or treated with the CXCR2 antagonist SB225002 (1ug/ml). (**C**) BrdU positive rate, indicating cell proliferation under each specified experimental condition. Note that cell proliferation decreased significantly both in the KD models and under treatment of cells with SB225002. Cell proliferation was remarkably lower in ACRP cells vs. A2780 cells in all events. Figure is of a representative experiment. Data was analyzed by one-way ANOVA. *p<0.05. N=3. Imagines were acquired in 10x magnification.

**Figure 6:**
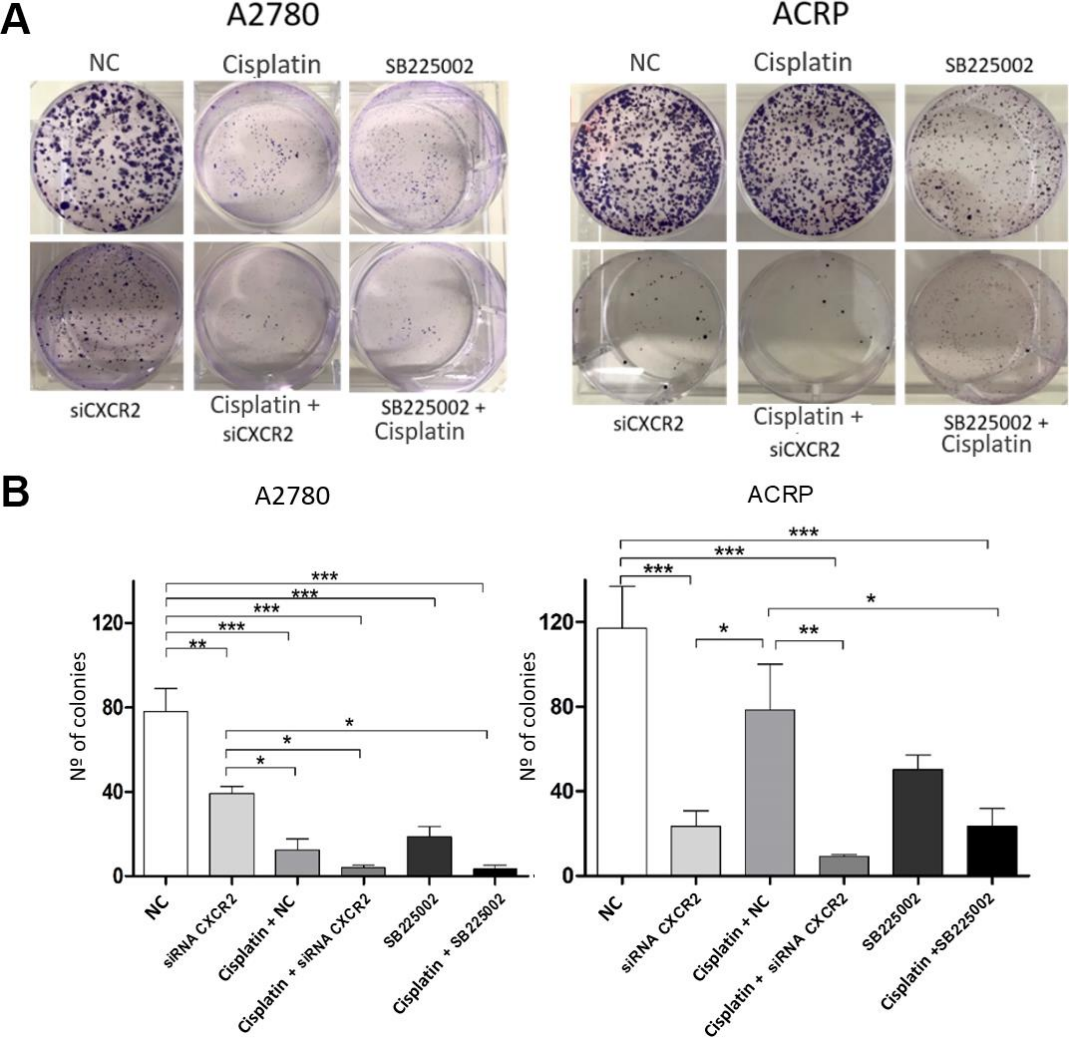
**CXCR2 is a pivotal molecule in OC cellular viability.** Clonogenic assays were run to evaluate the role of CXCR2 on OC cellular viability under the follow experimental conditions: i) cells transfected with empty vector (10μM), NC, control; ii) cells transfected with siRNA CXCR2 (10μM) to get the KD models; iii) cells that received cisplatin (5uM); iv) CXCR2 KD cells + cisplatin (5uM); v) cells treated with the CXCR2 antagonist SB225002 (1ug/ml); vi) cells that received combined therapy containing cisplatin (5uM) and SB225002 (1ug/ml). 150 cells of each lineage were plated on 6-well plates. Colonies were stained with crystal violet at D10. (**A**) Representative figure of the stained plate. The experiment confirmed A2780 sensitivity vs. ACRP resistance to cisplatin. A2780 CXCR2 KD cells were more viable than ACRP CXCR2 KD cells. Moreover, ACRP was more sensitive to SB225002 than A2780. On both cells, there was additive effects with the combined treatment containing cisplatin and siRNA against CXCR2 or cisplatin and SB225002. (**B**) Graphic representation of the percentage of colonies formed under each experimental condition, clearly reflecting Figure 5A. This experimental approach has proven the pivotal role of CXCR2 in the acquisition of cisplatin chemoresistant phenotype by OC cells. Data were analyzed by two-way ANOVA followed by Bonferroni post-test. *p<0.05, **p<0.01, ***p<0.001. N=3.

To further explore the relevance of CXCR2 expression in OC cells with and without cisplatin resistance, we analysed proliferation and cellular viability in CXCR2 KD cells. BrdU assay revealed that in both A2780 and ACRP, CXCR2 KD led to a significant decrease in cell proliferation, of approximately 2.0-fold and 3.5-fold, respectively (Figure 5A–5C) (p<0.05). Treatment of cells with SB225002, which is a CXCR2 inhibitor, also resulted in decreased proliferation in both cell lines (about 50% reduction; p<0.05). Cell proliferation was lower in ACRP vs. A2780 in all events, thus supporting the role of CXCR2 in OC progression with regard to chemoresistance. Next, we performed clonogenic assays to evaluate OC cellular viability (Figure 6A, 6B) in CXCR2 KD cells. A2780 sensitivity vs. ACRP resistance to cisplatin was confirmed. ACRP CXCR2 KD were more sensitive than A2780 KD (p<0.001). There was a synergistic effect in loss of cell viability when CXCR2 KD cells were treated with cisplatin (A2780 p<0.001; ACRP p<0.01) or cisplatin and SB225002 (A2780 p<0.001; ACRP p<0.01), these data being more prominent in ACRP cells. These findings indicate that CXCR2 plays a central role in the acquisition of cisplatin chemoresistant phenotype by OC cells.

### CXCR2 promotes *in vivo* OC tumor growth, angiogenesis and tumor invasion

**Figure 7:**
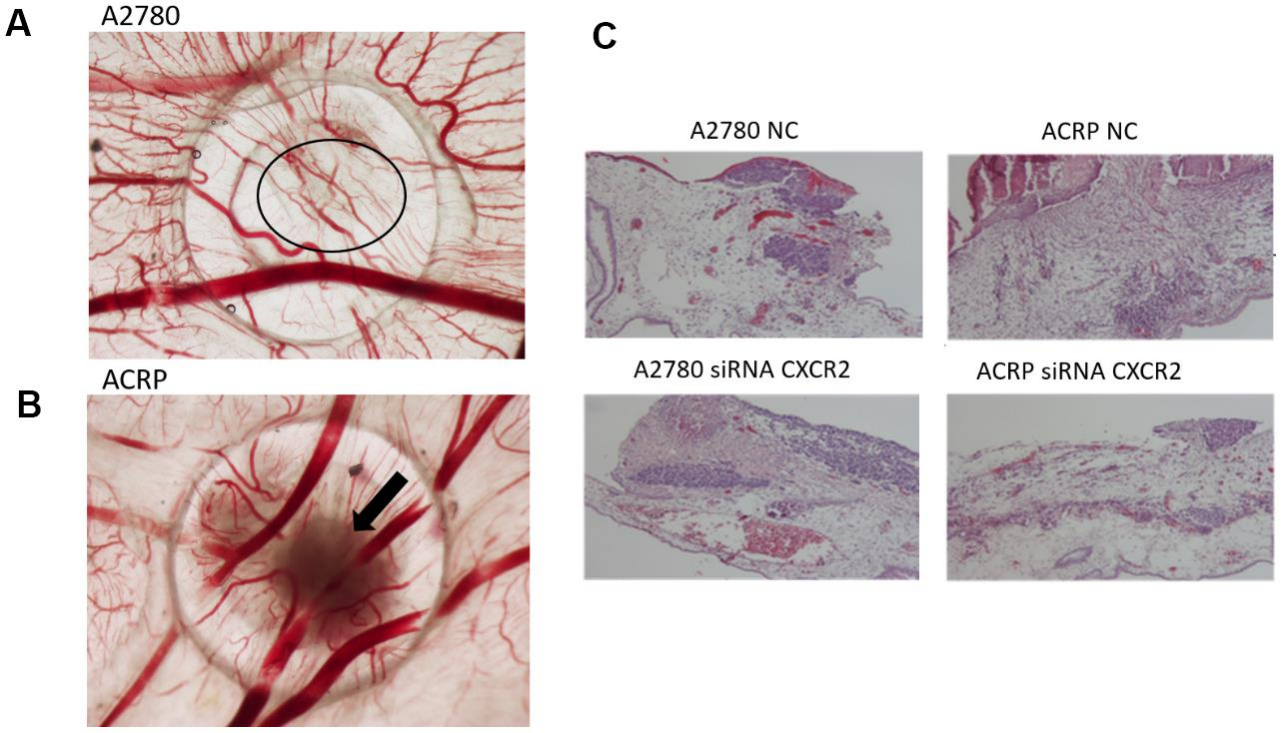
**CXCR2 promotes *in vivo* OC tumor growth (TG), angiogenesis and tumor invasion.** *In vivo* investigation of the role of CXCR2 on tumor growth, invasion and angiogenesis in OC was assessed by chicken embryo chorioallantoic membrane (CAM) method. (**A**) The number of new vessels formation with diameter lower than 20μm growing radially towards the ring area was counted in a blind fashion manner. No differences were noted between the lineages under the referred experimental condition. (**B**) Tumor size was measured at E10 egg inoculation with A2780 and ACRP OC cells, as: i) NC; ii) siRNA CXCR2 KD cells. Statistic significant difference were not observed probably due to number of replicates performed. However, when compared to A2780, TG was higher in ACRP than in A2780 cells. Moreover, CXCR2 KD prevented TG in later. The overall mortality rate for embryos/eggs was as expected (~10%). (**C**) The figure is also of a distinct invasion patterns between NC and CXCR2 KD ACRP and A2780 cells.

Investigation of the role of CXCR2 on OC tumor growth (TG) and angiogenesis was assessed by the chicken embryo chorioallantoic membrane (CAM) method. Tumor size was measured at E10 egg inoculated with A2780 or ACRP, NC and CXCR2 KD. When compared to A2780, TG was higher in ACRP. Moreover, CXCR2 KD prevented TG in ACRP (Figure 7A, 7B). There were no differences amongst cells with regard to angiogenesis and invasion (Figure 7A–7C). Statistic significant differences were not observed probably due to the number of replicates performed. However, our results point to a potential biological importance of CXCR2 in cisplatin resistant OC cells, inferred by continuous TG, which is partially, but significantly, reversed by CXCR2 KD.

### Silencing CXCR2 expression reduces EMT marker proteins SLUG and SNAIL in ACRP and seems to modulate PI3K/AKT/mTOR, but not MEK/ERK, pathway

**Figure 8:**
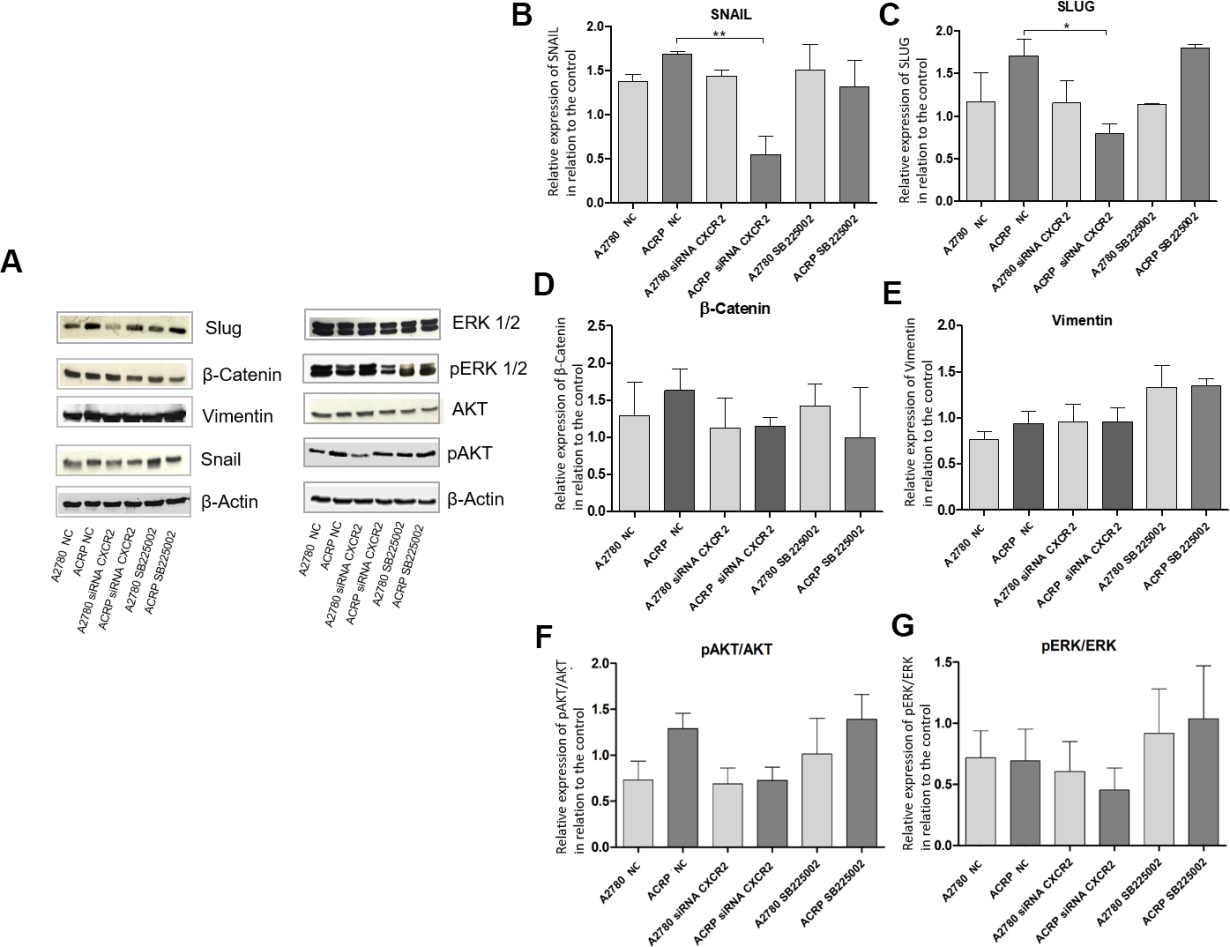
**Silencing CXCR2 expression reduces EMT marker proteins SLUG and SNAIL in ACRP and seems to modulate PI3K/AKT/mTOR, but not MEK/ERK, pathway.** Western blot assays were performed to investigate the expression of EMT marker proteins in ACRP and A2780 lines, as follow: i) CN cells (10; II) siRNA CXCR2 KD cells; iii) cells treated with SB225002. 50μ of protein were loaded into SDS-PAGE gels, proteins were separated by electrophoresis and blotted with the primary antibodies of interest. (**A**) Representative figure of the blots performed for each marker and different treatments, (**B**) SNAIL protein expression was significantly decreased in ACRP CXCR2 KD cells vs. ACRP wild type, but not in A2780 cells. (**C**) SLUG protein expression was significantly lower in ACRP CXCR2 KD cells with comparison to its wild-type counterpart, however not in A2780 cells. (**D**) β-Catenin and (**E**) Vimentin did not present significant statistical difference amongst the conditions studied. Moreover, when we seek to correlate pro-carcinogenic signaling pathways related to CXCR2, no statistic significant difference were noted both in (**F**) pAKT/AKT and (**G**) pERK/ERK pathways. It is worth note to address the biologic tendency of the CXCR2 KD models, but not the treatment of cells with SB225002, to inactivate PI3K/AKT/mTOR, but not MEK/ERK pathway (p= 0.7 and p=0.09 in ACRP and A2780, respectively). Data were analysed by two-way ANOVA followed the Bonferroni post- test. β-actin was used as a normalization control of the experiments. *p<0.022, **p<0.005. N=3.

Studies published so far have associated CXCR2 expression in cancer cells to the epithelial-mesenchymal transition (EMT) phenotype [25, 26]. Thus, we questioned whether this could also contribute to CXCR2-induced chemoresistance to cisplatin in OC cells. We analyzed protein expression of EMT markers as Snail, Slug and β-Catenin, and noted an increase in the expression of Snail and Slug in ACRP, which is reversed by CXCR2 KD (Figure 8B–8D). These data suggest that EMT is likely involved in CXCR2-dependent cisplatin chemoresistance in OC cells. These results were not observed when cells were treated with SB225002. In contrast, inhibition of CXCR2 did not significantly modulate vimentin nor reversed β-catenin expression in our study model (Figure 8D, 8E). We hypothesize that there might be a CXCR2-SNAIL-SLUG axis contributing to the CXCR2 role in cisplatin chemoresistance in OC cells. We, then, argued if silencing CXCR2 expression could modulate the classical carcinogenic signaling pathways PI3K/AKT/mTOR and MEK/ERK, leading to an observation of a biological tendency to decrease the expression of p-AKT (0.5-fold) in ACRP CXCR2 KD but not in A2780 KD (p=0.07 and *p*=0.09, respectively) (Figure 8F, 8G).

## DISCUSSION

In this study, we created an OC *in vitro* model of pan-resistant cells to cisplatin, paclitaxel and doxorubicin, thus reproducing a major challenge in fighting OC, which is recurrence of aggressive and lethal disease due to cisplatin chemoresistance. We further demonstrated that resistant cells depend on CXCR2 for survival and aggressiveness, at least partially through an EMT phenotype.

We aimed to elucidate the contribution of TME on chemoresistance motivated by our previous observations that ACRP cells secret CXCL2 and CXCL8 to their conditioned medium that ultimately modulate the fate of OC (manuscript in preparation). The present results suggest an autocrine effect of CXCL2/CXCL8 through CXCR2 expressed by OC cells. CXCL2 and CXCL8 have been correlated to cancer cells chemoresistance, migration, angiogenesis and progression in melanoma, colon and ovary cancers [19, 20, 27–29]. As previously mentioned, CXCL2 and CXCL8 depend on their specific binding to CXCR2 to elicit their cellular functions, a membrane receptor that has been detected in endothelial cells, infiltrating neutrophils, and tumor-associated macrophages, which also suggests an important regulatory role within the TME [30].

ACRP overexpresses CXCR2 in comparison to A2780. CXCR2 KD models were confirmed by the decrease of CXCR2 expression in both lineages when compared NC cells, being the effect more prominent in ACRP CXCR2 KD than in A2780 CXCR2 KD. The expression of CXCL2 and CXCL8 was significantly decreased in ACRP CXCR2 KD when compared to ACRP NC, but similar results were not observed in A2780. These results point to the occurrence of an intricate CXCR2/CXCL2-CXCL8 axis possibly modulating cisplatin resistance in OC and thus supporting the hypothesis of a role of the TME and a potential autocrine effect of chemokines on CXCR2 expressed by tumor cells.

Motivated by our results, we then questioned whether the cellular localization of CXCR2 could be altered in OC cells. Surprisingly, CXCR2 expression, which was significantly higher in ACRP than in A2780, was restricted to cellular nuclei instead of trafficking to the OC cells membrane. Although further studies are needed to elucidate the biological function of our findings, it is, at least to our knowledge, a novel observation. Nonetheless, recent data from the literatures cannot be neglected. DEK, an endogenous chromatin-binding factor that regulates hematopoiesis, can bind to CXCR2 and sequestrate it to the cellular nucleus. We hypothesized that binding of DEK to CXCR2 may, at least partially, justify the persistent nuclear expression of CXCR2 in the nuclei of OC cells, as DEK exerts its function in cellular nuclei in a CXCR2-dependent manner [31].

Overexpression of CXCR2 was previously associated with early recurrence of OC [24, 32]. In this study, information from 370 patients diagnosed with primary EOC was obtained from the TCGA database. Patients’ OS was opposed to CXCR2 overexpression in EOC cells, clearly demonstrating that CXCR2 is a marker of the disease poor prognosis. Similar experiments were run in intrahepatic, lung, cholangiocellular, pancreas, kidney, breast and colon cancer samples, also correlating poor disease prognosis to CXCR2 overexpression by cancer cells [22, 33–35].

Cell proliferation was lower in ACRP CXCR2 KD and in A2780 CXCR2 KD rather than when they were treated with SB225002, thus corroborating with the differential effects of CXCR2 KD and SB225002 treatment in OC cells and supporting the need to develop CXCR2 inhibitors that permeate cellular membrane to reach its target in cellular nuclei. The effect of CXCR2 on cell proliferation was shown in melanoma [36, 37] ovary [24] and prostate cancers [38, 39]. We found that A2780 was more sensitive to cisplatin than ACRP CXCR2 KD. Cellular viability of ACRP CXCR2 KD was lower than that of A2780. Our data show the greater effect of gene silencing than pharmacological intervention in ACRP, which suggest that resistant cells likely present intracellular expression of CXCR2, as proven in this work. Furthermore, cell viability decreased when CXCR2 was inhibited by SB225002 or CXCR2 KD in monotherapy. Moreover, there was synergy of these treatment approaches with cisplatin in both cell lines, being the effect more eminent in ACRP than A2780. A study conducted in OC demonstrated the synergic effect of the combined therapy containing sorafenib and SB225002 in inhibiting cell proliferation and angiogenesis [40]. In HGSOC cells SKOV3, CXCR2 KD comprised secretive activity of CXCL1 and CXCL8 [24] and, likewise our results, led to decreased cell viability, suggesting that silencing CXCR2 expression suppressed OC tumorigenicity *in vivo* and *in vitro.* Metastatic breast cancer cells can be re-sensitized to paclitaxel and doxorubicin by CXCR2 KD [41], as CXCR2 blockade was correlated to increased overall therapeutic response to antineoplastic substances, possibly due to lower TG and metastasis indexes [33, 41]. Significant inhibition of progression of CXCR2-negative metastatic lung cancer treated with paclitaxel was observed [33]. These findings are in agreement with ours through which CXCR2 emerge as a novel molecule orchestrating sensitivity of cancer cells to chemotherapy. Furthermore, CXCR2 arises as an unprecedented target to fight cancer in the adjuvant setting by overcoming chemotherapy resistance [33].

CXCR2-induced cell proliferation seems mediated by PI3K/AKT/mTOR and MEK/ERK pathways, which modulate cell cycle, apoptosis and angiogenesis, as well as the secretion of chemokines (as CXCL8) and cytokines (as seen for IL6) [Reviewed in 42]. The chemokine-regulated pathways led to cell adhesion, migration, chemotaxis, changes in cell morphology and regulation or activation of integrin [43, 44]. In our study model, though we did not find significant difference, one might suggest a biological tendency of CXCR2 to activate PI3K/AKT/mTOR, but not MEK/ERK pathway in OC cells.

Another important aspect to address is whether CXCR2 affects TG, angiogenesis and invasion of OC cells. CXCR2 stimulated TG, invasion and metastasis in murine KRAS/p53-mutant lung adenocarcinoma cell line [22]. In contrast, CXCR2 seems to induce TG in an angiogenesis-independent fashion in ACRP, however not in A2780. Further experiments are necessary to confirm this hypothesis.

EMT has been implicated as a key process involved in tumor invasion and metastasis, affecting characteristics such as stemness, apoptosis and immune system [45]. Snail, an EMT marker, promoted CXCR2 ligand-dependent tumor progression in lung cancer [46]. Moreover, EMT was directly related to chemoresistance [47–49]. Our results showed overexpression of EMT markers, such as Snail and Slug, in ACRP vs. A2780. We reported that ACRP overexpressed Snail, Slug and β-catenin. Nevertheless, only overexpression of Snail and Slug were reversed in ACRP CXCR2 KD. Snail is a transcriptional repressor of E-cadherin and induces trafficking of myeloid-derived suppressor cells via upregulation of CXCR2. *In silico* analysis of EOC data obtained from TCGA database indicated that Snail is correlated with the secretion of CXCL2/CXCL8 to TME [49, 50], thus revealing that Snail has multiple important functions, including modulation of the immune system and EMT.

In conclusion, our study proves that CXCR2 is retained in the nucleus of OC cells that acquired cisplatin resistant phenotype, being correlated with poor prognosis of the disease and its high mortality rate in patients. In addition, CXCR2 is associated with tumor proliferation and growth in OC resistant cells, showing its role in disease chemoresistant phenotype acquisition and progression. Thus, effective strategies through the synthesis of highly lipophilic analogous molecules of the prototype CXCR2 competitive inhibitor SB225002 can inhibit CXCR2 carcinogenic role in OC, in an economical and clinical viable fashion. In any event, we, herein, introduce a novel mechanism that contributes to chemoresistance of OC cells to cisplatin.

Our study has proven that the inhibition of CXCR2 pathway may not only lead to OC antitumor properties but may also act as a chemosensitizer of tumor cells to cisplatin. In summary, our results present innovative strategy to treat pan-chemoresistant OC, by inhibiting the persistent and anomalous nuclear overexpression of CXCR2 in cisplatin resistant disease, therefore, opening a novel avenue to combat this still highly deadly disease.

## MATERIALS AND METHODS

### Cell lines and culture conditions

Pan-resistant ACRP cells were generated from its parental counterpart A2780 lineage, following chronic exposure to cisplatin (1μM to 10 μM). Chemoresistance was verified by the MTT method [51] through the calculation of estimated IC_50_ of cisplatin, paclitaxel and doxorubicin. Lineages were cultured in complete DMEM medium (Invitrogen) supplemented with FBS 10%(v/v), penicillin/streptomycin1%(w/v), amphotericin1%(w/v), at 37° C in atmosphere of 5% CO_2_.

### Generation of CXCR2 KD cells

CXCR2 gene expression was silenced using small interfering siRNACXCR2 plasmid (10 μM), 5μl of lipofectamine 2000 in 125μl of Opti-MEM® Reduced-Serum Medium, as manufacturer suggestion (Invitrogen). Control experiments were run in parallel, using Stealth RNAi siRNA Negative Control Duplex (10μM) (Invitrogen). CXCR2 was also pharmacologically inhibited by SB225002 (1ug/mL) (Abcam) diluted in 0.001%(v/v) dimethylsulfoxide (DMSO).

### RNA extraction and real-time reverse transcription polymerase chain reaction (qRT-PCR)

RNA was extracted using TRIzol (Invitrogen), following the manufacturer instructions. cDNA was obtained by SuperScript First-Strand Synthesis System (Invitrogen; manufacturer protocol). For quantitative q-RT-PCR reactions, 50ng cDNA were amplified in SYBR Green PCR Master Mix (Applied Biosystems). Gene expression was annotated as 2^-^ΔΔ^C^, using ABI Prism 7500 Fast System software (Applied Biosystems). Amplification conditions were 95° C for DNA denaturation, melting temperature 58° C and 72° C for DNA extension; 40 cycles. Primers were: CXCR2: F3’TTGCAACCCAGGTCAGAAGTT5’ (10μm), R3’CAGCTGTGACCTGCTGTTATT5’ (10μm);

GAPDH: F3’CAGCCTCAAGATCATCAGCA5’ (10μm), R3’ACAGTCTTCTGGGTGGCAGT5’ (10μm) (Invitrogen).

### Clonogenic assay

CXCR-modulated cell viability was investigated in CXCR2 KD cells or lineages treated SB225002 (1μg/mL) (150 cells/well). Cells were harvested at D10 cisplatin treatment (5μM). Colony formation was analysed by crystal violet staining [52]. Experimental controls were done using empty siRNA plasmids or wells containing only DMSO 0.001%(v/v).

### Western blot

EMT markers, PI3K/AKT/mTOR and MEK/ERK signalling pathways elements were analysed by Western blot. Total proteins were extracted from cells using RIPA buffer (NaCl 1%w/v, sodium deoxycholate 0.5%w/v, SDS 0.1%w/v), Tris (50mM; pH 8.00). 30μg of protein were applied to 10%(w/v) polyacrylamide gels, separated by SDS-PAGE electrophoresis, then transferred to PVDF membranes (Millipore, USA). Membranes were blocked with skim milk 5%(w/v) or BSA 5%(w/v) for 30 minutes, then incubated overnight with primary antibody (1:1000) at 4° C. Anti-β-actin (1:4000) was used as internal control for semi-quantitative analysis. After incubation with secondary antibody (60 minutes), at room temperature (RT), blots were revealed with ECL reagent (manufacturer protocol) (GE). Protein expression was analysed by Lab software 6.1 version for Windows (Bio Rad).

### Immunofluorescence

Cells were fixed on glass slides with methanol 20%(v/v), embedded in HCL 2M for 30 minutes at RT, incubated with anti-BrdU (1:10) or anti-CXCR2 (1:200) (Abcam) for 60 minutes at RT, then incubated with Alexa Fluor 494 goat anti-mouse or Alexa Fluor 494 goat anti-rabbit secondary antibodies (1:100) (Invitrogen) for 60 minutes, at RT, in the dark. Cells nuclei were stained with DAPI (1:10) (Thermo Fisher) for 15 minutes at RT. Images were acquired in Zeiss Z1 apotome microscope (LEICA) at 10x magnification.

### Chicken embryo chorioallantoic membrane (CAM) angiogenesis and tumor growth assay

Chicken embryo chorioallantoic membrane (CAM) method was used to evaluate angiogenesis and tumor growth (TG) [53, 54]. Fertilized *Gallus gallus* eggs were incubated horizontally at 37.8° C in a humidified atmosphere (embryonic day; E). On E3, a square window was opened on the shell after removal of 2-2.5mL of albumen. Window was sealed and eggs were returned to incubator. At E10, 10^6^ cells were placed into a 3 mm silicon ring, under sterile conditions, on top of growing CAM. Eggs were re-sealed and returned to incubator for 4 days. After removing the ring, CAM was excised from embryos, photographed under stereoscope at 20x magnification (Olympus, SZX16 coupled with DP71 camera). The number of new vessels (less than 20 μm diameter) growing radially towards the ring area was counted in a blind fashion manner, as well as the observation of tumor growth and invasion.

### Survival analysis

*In silico* analysis was performed to correlate EOC patients’ overall survival rate (OS) with CXCR2 expression from 370 patients diagnosed with primary EOC were extract from The Cancer Genome Atlas Program (TCGA Computational Tools) [55].

### Statistical analysis

Results are presented as mean ± SD. Statistical significance was calculated by unpaired *t*-Student test, one-way ANOVA or two-way ANOVA followed Bonferroni *post-hoc* test, as indicated in Figure legends (GraphPad Prism software version 5.00 for Windows). *In silico* patients’ OS analysis was expressed as Kaplan-Meier curves. *p*<0.05 was considered for statistical significance.

## References

[1] Bray F, Ferlay J, Soerjomataram I, Siegel RL, Torre LA, Jemal A. Global cancer statistics 2018: GLOBOCAN estimates of incidence and mortality worldwide for 36 cancers in 185 countries. CA Cancer J Clin. 2018; 68:394–424. 10.3322/caac.21492 30207593

[2] Stats fact sheets: ovary cancer. National Cancer Institute. 2019. https://seer.cancer.gov/statfacts/html/ovary.

[3] Prat J. Ovarian carcinomas: five distinct diseases with different origins, genetic alterations, and clinicopathological features. Virchows Arch. 2012; 460:237–49. 10.1007/s00428-012-1203-5 22322322

[4] Karnezis AN, Cho KR, Gilks CB, Pearce CL, Huntsman DG. The disparate origins of ovarian cancers: pathogenesis and prevention strategies. Nat Rev Cancer. 2017; 17:65–74. 10.1038/nrc.2016.113 27885265

[5] Kim J, Park EY, Kim O, Schilder JM, Coffey DM, Cho CH, Bast RC Jr. Cell Origins of High-Grade Serous Ovarian Cancer. Cancers (Basel). 2018; 10:433. 10.3390/cancers10110433 30424539 PMC6267333

[6] Labidi-Galy SI, Papp E, Hallberg D, Niknafs N, Adleff V, Noe M, Bhattacharya R, Novak M, Jones S, Phallen J, Hruban CA, Hirsch MS, Lin DI, et al. High grade serous ovarian carcinomas originate in the fallopian tube. Nat Commun. 2017; 8:1093. 10.1038/s41467-017-00962-1 29061967 PMC5653668

[7] Kurman RJ, Shih IeM. The Dualistic Model of Ovarian Carcinogenesis: Revisited, Revised, and Expanded. Am J Pathol. 2016; 186:733–47. 10.1016/j.ajpath.2015.11.011 27012190 PMC5808151

[8] Shih IeM, Kurman RJ. Ovarian tumorigenesis: a proposed model based on morphological and molecular genetic analysis. Am J Pathol. 2004; 164:1511–18. 10.1016/s0002-9440(10)63708-x 15111296 PMC1615664

[9] Patch AM, Christie EL, Etemadmoghadam D, Garsed DW, George J, Fereday S, Nones K, Cowin P, Alsop K, Bailey PJ, Kassahn KS, Newell F, Quinn MC, et al. Whole-genome characterization of chemoresistant ovarian cancer. Nature. 2015; 521:489–94. 10.1038/nature14410 26017449

[10] Teer JK, Yoder S, Gjyshi A, Nicosia SV, Zhang C, Monteiro AN. Mutational heterogeneity in non-serous ovarian cancers. Sci Rep. 2017; 7:9728. 10.1038/s41598-017-10432-9 28852190 PMC5574976

[11] Ahmed AA, Etemadmoghadam D, Temple J, Lynch AG, Riad M, Sharma R, Stewart C, Fereday S, Caldas C, Defazio A, Bowtell D, Brenton JD. Driver mutations in TP53 are ubiquitous in high grade serous carcinoma of the ovary. J Pathol. 2010; 221:49–56. 10.1002/path.2696 20229506 PMC3262968

[12] Ho ES, Lai CR, Hsieh YT, Chen JT, Lin AJ, Hung MH, Liu FS. p53 mutation is infrequent in clear cell carcinoma of the ovary. Gynecol Oncol. 2001; 80:189–93. 10.1006/gyno.2000.6025 11161858

[13] Kuo KT, Mao TL, Jones S, Veras E, Ayhan A, Wang TL, Glas R, Slamon D, Velculescu VE, Kuman RJ, Shih IeM. Frequent activating mutations of PIK3CA in ovarian clear cell carcinoma. Am J Pathol. 2009; 174:1597–601. 10.2353/ajpath.2009.081000 19349352 PMC2671248

[14] Cancer Genome Atlas Research Network. Integrated genomic analyses of ovarian carcinoma. Nature. 2011; 474:609–15. 10.1038/nature10166 21720365 PMC3163504

[15] Bonome T, Lee JY, Park DC, Radonovich M, Pise-Masison C, Brady J, Gardner GJ, Hao K, Wong WH, Barrett JC, Lu KH, Sood AK, Gershenson DM, et al. Expression profiling of serous low malignant potential, low-grade, and high-grade tumors of the ovary. Cancer Res. 2005; 65:10602–12. 10.1158/0008-5472.CAN-05-2240 16288054

[16] Bast RC Jr, Hennessy B, Mills GB. The biology of ovarian cancer: new opportunities for translation. Nat Rev Cancer. 2009; 9:415–28. 10.1038/nrc2644 19461667 PMC2814299

[17] Paes MF, Daltoé RD, Madeira KP, Rezende LC, Sirtoli GM, Herlinger AL, Souza LS, Coitinho LB, Silva D, Cerri MF, Chiaradia AC, Carvalho AA, Silva IV, Rangel LB. A retrospective analysis of clinicopathological and prognostic characteristics of ovarian tumors in the State of Espírito Santo, Brazil. J Ovarian Res. 2011; 4:14. 10.1186/1757-2215-4-14 21827671 PMC3163211

[18] Gao M, Herlinger AL, Wu R, Wang TL, Shih IM, Kong B, Rangel LB, Yang JM. NAC1 attenuates BCL6 negative autoregulation and functions as a BCL6 coactivator of FOXQ1 transcription in cancer cells. Aging (Albany NY). 2020; 12:9275–91. 10.18632/aging.103203 32412910 PMC7288929

[19] Gabellini C, Trisciuoglio D, Desideri M, Candiloro A, Ragazzoni Y, Orlandi A, Zupi G, Del Bufalo D. Functional activity of CXCL8 receptors, CXCR1 and CXCR2, on human malignant melanoma progression. Eur J Cancer. 2009; 45:2618–27. 10.1016/j.ejca.2009.07.007 19683430

[20] Baier PK, Wolff-Vorbeck G, Eggstein S, Baumgartner U, Hopt UT. Cytokine expression in colon carcinoma. Anticancer Res. 2005; 25:2135–39. 16158955

[21] Ohri CM, Shikotra A, Green RH, Waller DA, Bradding P. Chemokine receptor expression in tumour islets and stroma in non-small cell lung cancer. BMC Cancer. 2010; 10:172. 10.1186/1471-2407-10-172 20429924 PMC2876080

[22] Saintigny P, Massarelli E, Lin S, Ahn YH, Chen Y, Goswami S, Erez B, O’Reilly MS, Liu D, Lee JJ, Zhang L, Ping Y, Behrens C, et al. CXCR2 expression in tumor cells is a poor prognostic factor and promotes invasion and metastasis in lung adenocarcinoma. Cancer Res. 2013; 73:571–82. 10.1158/0008-5472.CAN-12-0263 23204236 PMC3548940

[23] Boisvert WA, Rose DM, Johnson KA, Fuentes ME, Lira SA, Curtiss LK, Terkeltaub RA. Up-regulated expression of the CXCR2 ligand KC/GRO-alpha in atherosclerotic lesions plays a central role in macrophage accumulation and lesion progression. Am J Pathol. 2006; 168:1385–95. 10.2353/ajpath.2006.040748 16565511 PMC1606562

[24] Yang G, Rosen DG, Liu G, Yang F, Guo X, Xiao X, Xue F, Mercado-Uribe I, Huang J, Lin SH, Mills GB, Liu J. CXCR2 promotes ovarian cancer growth through dysregulated cell cycle, diminished apoptosis, and enhanced angiogenesis. Clin Cancer Res. 2010; 16:3875–86. 10.1158/1078-0432.CCR-10-0483 20505188 PMC2930833

[25] Bates RC, DeLeo MJ 3rd, Mercurio AM. The epithelial-mesenchymal transition of colon carcinoma involves expression of IL-8 and CXCR-1-mediated chemotaxis. Exp Cell Res. 2004; 299:315–24. 10.1016/j.yexcr.2004.05.033 15350531

[26] Henriques TB, Santos DZ, Hakeem-Sanni MF, Silva IV, Azevedo Rangel LB. The CXCR2/SNAIL Axis: Is this a novel anti- tumor therapeutical target for cancer cells undergoing Epithelial-Mesenchimal transition process? J. J Carcinog Mutagen. 2020; 11:351.

[27] Infanger DW, Cho Y, Lopez BS, Mohanan S, Liu SC, Gursel D, Boockvar JA, Fischbach C. Glioblastoma stem cells are regulated by interleukin-8 signaling in a tumoral perivascular niche. Cancer Res. 2013; 73:7079–89. 10.1158/0008-5472.CAN-13-1355 24121485 PMC3880850

[28] Chin AR, Wang SE. Cytokines driving breast cancer stemness. Mol Cell Endocrinol. 2014; 382:598–602. 10.1016/j.mce.2013.03.024 23562748

[29] Stadtmann A, Zarbock A. CXCR2: From Bench to Bedside. Front Immunol. 2012; 3:263. 10.3389/fimmu.2012.00263 22936934 PMC3426767

[30] Acharyya S, Oskarsson T, Vanharanta S, Malladi S, Kim J, Morris PG, Manova-Todorova K, Leversha M, Hogg N, Seshan VE, Norton L, Brogi E, Massagué J. A CXCL1 paracrine network links cancer chemoresistance and metastasis. Cell. 2012; 150:165–78. 10.1016/j.cell.2012.04.042 22770218 PMC3528019

[31] Capitano ML, Mor-Vaknin N, Saha AK, Cooper S, Legendre M, Guo H, Contreras-Galindo R, Kappes F, Sartor MA, Lee CT, Huang X, Markovitz DM, Broxmeyer HE. Secreted nuclear protein DEK regulates hematopoiesis through CXCR2 signaling. J Clin Invest. 2019; 129:2555–70. 10.1172/JCI127460 31107242 PMC6546479

[32] Bolitho C, Hahn MA, Baxter RC, Marsh DJ. The chemokine CXCL1 induces proliferation in epithelial ovarian cancer cells by transactivation of the epidermal growth factor receptor. Endocr Relat Cancer. 2010; 17:929–40. 10.1677/ERC-10-0107 20702723

[33] Sharma B, Nawandar DM, Nannuru KC, Varney ML, Singh RK. Targeting CXCR2 enhances chemotherapeutic response, inhibits mammary tumor growth, angiogenesis, and lung metastasis. Mol Cancer Ther. 2013; 12:799–808. 10.1158/1535-7163.MCT-12-0529 23468530 PMC3653628

[34] Sueoka H, Hirano T, Uda Y, Iimuro Y, Yamanaka J, Fujimoto J. Blockage of CXCR2 suppresses tumor growth of intrahepatic cholangiocellular carcinoma. Surgery. 2014; 155:640–49. 10.1016/j.surg.2013.12.037 24582495

[35] Qiao B, Luo W, Liu Y, Wang J, Liu C, Liu Z, Chen S, Gu J, Qi X, Wu T. The prognostic value of CXC chemokine receptor 2 (CXCR2) in cancers: a meta-analysis. Oncotarget. 2017; 9:15068–76. 10.18632/oncotarget.23492 29599927 PMC5871098

[36] Singh S, Sadanandam A, Nannuru KC, Varney ML, Mayer-Ezell R, Bond R, Singh RK. Small-molecule antagonists for CXCR2 and CXCR1 inhibit human melanoma growth by decreasing tumor cell proliferation, survival, and angiogenesis. Clin Cancer Res. 2009; 15:2380–86. 10.1158/1078-0432.CCR-08-2387 19293256 PMC4232212

[37] Singh S, Varney M, Singh RK. Host CXCR2-dependent regulation of melanoma growth, angiogenesis, and experimental lung metastasis. Cancer Res. 2009; 69:411–15. 10.1158/0008-5472.CAN-08-3378 19147552 PMC2652477

[38] Maxwell PJ, Gallagher R, Seaton A, Wilson C, Scullin P, Pettigrew J, Stratford IJ, Williams KJ, Johnston PG, Waugh DJ. HIF-1 and NF-kappaB-mediated upregulation of CXCR1 and CXCR2 expression promotes cell survival in hypoxic prostate cancer cells. Oncogene. 2007; 26:7333–45. 10.1038/sj.onc.1210536 17533374

[39] Murphy C, McGurk M, Pettigrew J, Santinelli A, Mazzucchelli R, Johnston PG, Montironi R, Waugh DJ. Nonapical and cytoplasmic expression of interleukin-8, CXCR1, and CXCR2 correlates with cell proliferation and microvessel density in prostate cancer. Clin Cancer Res. 2005; 11:4117–27. 10.1158/1078-0432.CCR-04-1518 15930347

[40] Devapatla B, Sharma A, Woo S. CXCR2 Inhibition Combined with Sorafenib Improved Antitumor and Antiangiogenic Response in Preclinical Models of Ovarian Cancer. PLoS One. 2015; 10:e0139237. 10.1371/journal.pone.0139237 26414070 PMC4587670

[41] Sharma B, Varney ML, Saxena S, Wu L, Singh RK. Induction of CXCR2 ligands, stem cell-like phenotype, and metastasis in chemotherapy-resistant breast cancer cells. Cancer Lett. 2016; 372:192–200. 10.1016/j.canlet.2015.12.011 26797460 PMC4821546

[42] Wang B, Hendricks DT, Wamunyokoli F, Parker MI. A growth-related oncogene/CXC chemokine receptor 2 autocrine loop contributes to cellular proliferation in esophageal cancer. Cancer Res. 2006; 66:3071–77. 10.1158/0008-5472.CAN-05-2871 16540656

[43] Baggiolini M. Chemokines and leukocyte traffic. Nature. 1998; 392:565–68. 10.1038/33340 9560152

[44] Zarbock A, Ley K. Mechanisms and consequences of neutrophil interaction with the endothelium. Am J Pathol. 2008; 172:1–7. 10.2353/ajpath.2008.070502 18079440 PMC2189633

[45] Liang L, Sun H, Zhang W, Zhang M, Yang X, Kuang R, Zheng H. Meta-Analysis of EMT Datasets Reveals Different Types of EMT. PLoS One. 2016; 11:e0156839. 10.1371/journal.pone.0156839 27258544 PMC4892621

[46] Yanagawa J, Walser TC, Zhu LX, Hong L, Fishbein MC, Mah V, Chia D, Goodglick L, Elashoff DA, Luo J, Magyar CE, Dohadwala M, Lee JM, et al. Snail promotes CXCR2 ligand-dependent tumor progression in non-small cell lung carcinoma. Clin Cancer Res. 2009; 15:6820–29. 10.1158/1078-0432.CCR-09-1558 19887480 PMC2783274

[47] Peinado H, Olmeda D, Cano A. Snail, Zeb and bHLH factors in tumour progression: an alliance against the epithelial phenotype? Nat Rev Cancer. 2007; 7:415–28. 10.1038/nrc2131 17508028

[48] Qiu WZ, Zhang HB, Xia WX, Ke LR, Yang J, Yu YH, Liang H, Huang XJ, Liu GY, Li WZ, Xiang YQ, Kang TB, Guo X, Lv X. The CXCL5/CXCR2 axis contributes to the epithelial-mesenchymal transition of nasopharyngeal carcinoma cells by activating ERK/GSK-3β/snail signalling. J Exp Clin Cancer Res. 2018; 37:85. 10.1186/s13046-018-0722-6 29665837 PMC5905166

[49] Taki M, Abiko K, Baba T, Hamanishi J, Yamaguchi K, Murakami R, Yamanoi K, Horikawa N, Hosoe Y, Nakamura E, Sugiyama A, Mandai M, Konishi I, Matsumura N. Snail promotes ovarian cancer progression by recruiting myeloid-derived suppressor cells via CXCR2 ligand upregulation. Nat Commun. 2018; 9:1685. 10.1038/s41467-018-03966-7 29703902 PMC5923228

[50] Stemmer V, de Craene B, Berx G, Behrens J. Snail promotes Wnt target gene expression and interacts with beta-catenin. Oncogene. 2008; 27:5075–80. 10.1038/onc.2008.140 18469861

[51] Riss TL, Moravec RA, Niles AL, Duellman S, Benink HA, Worzella TJ, Minor L. Cell Viability Assays. 2013 May 1 [updated 2016 Jul 1]. In: Markossian S, Sittampalam GS, Grossman A, Brimacombe K, Arkin M, Auld D, Austin CP, Baell J, Caaveiro JMM, Chung TDY, Coussens NP, Dahlin JL, Devanaryan V, et al. editors. Assay Guidance Manual. Bethesda (MD): Eli Lilly and Company and the National Center for Advancing Translational Sciences; 2004.

[52] Franken NA, Rodermond HM, Stap J, Haveman J, van Bree C. Clonogenic assay of cells *in vitro*. Nat Protoc. 2006; 1:2315–19. 10.1038/nprot.2006.339 17406473

[53] Estrada MF, Rebelo SP, Davies EJ, Pinto MT, Pereira H, Santo VE, Smalley MJ, Barry ST, Gualda EJ, Alves PM, Anderson E, Brito C. Modelling the tumour microenvironment in long-term microencapsulated 3D co-cultures recapitulates phenotypic features of disease progression. Biomaterials. 2016; 78:50–61. 10.1016/j.biomaterials.2015.11.030 26650685

[54] Prazeres H, Torres J, Rodrigues F, Pinto M, Pastoriza MC, Gomes D, Cameselle-Teijeiro J, Vidal A, Martins TC, Sobrinho-Simões M, Soares P. Chromosomal, epigenetic and microRNA-mediated inactivation of LRP1B, a modulator of the extracellular environment of thyroid cancer cells. Oncogene. 2011; 30:1302–17. 10.1038/onc.2010.512 21057533

[55] The Cancer Genome Atlas Program. National Cancer Institute. 2019. https://www.cancer.gov/about-nci/organization/ccg/research/structural-genomics/tcga

